# Direct frequency comb optical frequency standard based on two-photon transitions of thermal atoms

**DOI:** 10.1038/srep15114

**Published:** 2015-10-13

**Authors:** S. Y. Zhang, J. T. Wu, Y. L. Zhang, J. X. Leng, W. P. Yang, Z. G. Zhang, J. Y. Zhao

**Affiliations:** 1 Department of Electronics, School of Electronics Engineering and Computer Science, Peking University, Beijing, 100871, China

## Abstract

Optical clocks have been the focus of science and technology research areas due to their capability to provide highest frequency accuracy and stability to date. Their superior frequency performance promises significant advances in the fields of fundamental research as well as practical applications including satellite-based navigation and ranging. In traditional optical clocks, ultrastable optical cavities, laser cooling and particle (atoms or a single ion) trapping techniques are employed to guarantee high stability and accuracy. However, on the other hand, they make optical clocks an entire optical tableful of equipment, and cannot work continuously for a long time; as a result, they restrict optical clocks used as very convenient and compact time-keeping clocks. In this article, we proposed, and experimentally demonstrated, a novel scheme of optical frequency standard based on comb-directly-excited atomic two-photon transitions. By taking advantage of the natural properties of the comb and two-photon transitions, this frequency standard achieves a simplified structure, high robustness as well as decent frequency stability, which promise widespread applications in various scenarios.

With the development of optical clocks^1^,^2^,^3^, extraordinary results in fundamental research^4^,^5^ as well as practical applications including satellite-based navigation and ranging^6^ have been demonstrated in the last decade. Benefited by the development of frequency-stabilization techniques (ultrastable optical cavities)^7^,^8^,^9^ for continuous-wave lasers and optical frequency combs^10^,^11^,^12^ , traditional optical clocks based on optical transitions in ultra-cold neutral atoms and trapped ions^2^,^13^,^14^ have achieved a great advance in optical frequency standards. As a result, the state-of-the-art optical clock has reached 10^−18^ level both in accuracy and stability^3^. However, in the meantime, ultrastable optical cavities, laser cooling and particle trapping techniques are susceptible to environments, which hinder the widespread use of optical clocks in many practical applications.

In a conventional optical clock scheme, a pre-stabilised continuous wave laser with narrow linewidth is employed as the probe laser to excite the narrow clock transitions of either a single ion or neutral toms. To prevent Doppler effects, collision shift and other motion induced disturbances to the clock transition resonances, it is necessary to use laser-cooling and particle-trapping technologies, such as magneto-optical trap and ion trap, to laser-cool the atoms and confine their motion into the Lamb-Dicke regime^2^,^14^. On the other hand, the probe laser is required to be pre-stabilised to a high-finesses optical reference cavity for linewidth narrowing so as to achieve precise transition resonance probing and laser locking^8^,^9^. Furthermore, an optical frequency comb operates as the clockwork to faithfully transfer the high stability and accuracy of the optical standard to countable microwave frequency^1^,^15^.

Much attention of the optical clock research is paid on pushing the accuracy and stability to the quantum limit^16^. Hence, novel material of optical cavity with high elastic modulus and large conductivity is explored to narrow the laser linewidth^8^. Moreover, technologies for particle cooling and trapping, e.g. optical lattice with magic wavelength cooling, have been continuously improved to obtain narrower and more stable transition line. All these efforts enable uncertainty and instability of state-of-the-art optical clock^3^ to reach the order of 10^−18^. However, these devices related to high stable optical cavity, laser cooling and particle trapping usually make the optical clocks occupy large size. Meanwhile, they are highly susceptible to environmental influences, including acoustic noise, mechanical vibrations, temperature drifts, etc. These entire characteristics make optical clocks cannot work continuously for a long time, and restrict the use of optical clocks in various application scenarios beyond the research labs. Until now, the time-keeping clocks used for Coordinated Universal Time (UTC) are still microwave clocks (like Microsemi 5071A), whose stability is much worse than that of optical clocks.

The orientation of this article is to explore a simplified and robust scheme of optical frequency standard that could expedite practical and commercial applications, and ultimately to make a time-keeping optical clock. To achieve this goal, this scheme utilizes the optical frequency comb, instead of the continuous wave laser in conventional scheme, as the probe laser to directly excite the atomic clock transitions, and finally frequency locks one of the comb lines to the centre of transition resonance. With the optical comb simultaneously operating as the probe laser as well as the perfect frequency stability transfer gear between optical and microwave domains, this scheme omits the continuous wave probe laser and relevant inter-laser frequency link servos, which are essential parts in conventional optical clocks. Moreover, in contrast to continuous wave lasers, optical combs have naturally narrow linewidth, usually about kilohertz^17^ with simple optimizing, and even on the order of subhertz^18^,^19^,^20^ after stabilised to similarly narrow references. This natural advantage enables the direct frequency comb optical frequency standards to avoid the use of delicate optical cavities.

In view of the large amount of comb lines in a frequency comb, each of comb lines shares rather low power (usually on the order of microwatts), which is insufficient for effective atomic transition excitation. To obtain high signal-to-noise ratio (SNR) of transition resonance probing, it is preferable that more comb lines could contribute to the transition excitations. With regard to this requirement, two-photon transition^21^ (TPT) would serve as an superior candidate for direct frequency comb optical frequency standards, where, as illustrated in Fig. 1a, a series of comb line pairs with the same sum frequency can jointly participate in the transition excitation once the resonance condition is fulfilled^22^. The TPTs spectroscopy has been a research hotspot over the past four decades^21^,^22^,^23^,^24^,^25^,^26^. With the development of the frequency comb, the direct frequency comb spectroscopy (DFCS) on the TPTs is still an immediate area of research focus^27^,^28^,^29^,^30^,^31^. Thanks to the perfect match between the two-photon resonance condition and the equally spaced line structure of the comb, all comb lines near the virtual level are involved, leading to a high degree of transition excitation. Furthermore, TPTs excited by two counter-propagating lasers (Fig. 1b) possess the natural immunity to Doppler effects—the motion-induced Doppler frequency shifts of two photons dramatically cancel out, resulting in a Doppler-free transition line^23^,^24^. This intrinsic Doppler-free property enables us to extract relatively narrow transition lines from a thermal vapour atomic system, which liberates the atoms from laser-cooling and atom-trapping systems. Besides, one can achieve an atom density of about 3.5 × 10^17^/m^3^ at 60 °C temperature (measured by VICTOR DM6801A + with 0.1−°C resolution and 0.2% accuracy) of the thermal Rb atoms, which is usually more than one order of magnitude higher than that in the cold atomic system. As a result, it can realize higher SNR and good robustness in the thermal atomic system.

As described above, in this direct frequency comb optical frequency standard scheme, the laser system and atomic system are simplified to just an optical comb and a thermal vapour cell, respectively, while the intrinsic properties of the comb and TPTs ensure a relatively narrow laser linewidth and decent immunity to Doppler effects, which to some extent replace the roles of ultrastable optical cavity and laser-cooling systems. Following this framework, in this article, we proposed, and experimentally demonstrated, a novel scheme of optical frequency standard based on comb-directly-excited atomic two-photon transitions. By taking advantage of the natural properties of the comb and two-photon transitions, this frequency standard achieves a simplified structure, high robustness as well as decent frequency stability, which promise widespread applications in various scenarios.

In this experiment, the rubidium (Rb) 5S_1/2_ (F = 2) → 5D_5/2_ (F = 4) TPT with 5P energy level as the intermediate state (Fig. 1a) was employed as the frequency transition, which has an excitation wavelength near 778 nm and a linewidth of 300 kHz (corresponding to 240 ns lifetime of the 5D level). We adopted a frequency-doubled Er-fibre-based comb as the probe laser in consideration of the superiorities of fibre-based comb in flexibility, robustness and cost. Each particular mode of the comb can be expressed as ν_*n*_ = *f*_0_ + *nf*_rep_, where *f*_0_ is the carrier-envelop offset frequency, *f*_rep_ is the repetition rate, and *n* is an integer on the order of 10^6^. To obtain a fully stabilised *f*_rep_ (as the clock microwave output), we frequency locked both ν_*n*_ and *f*_0_, directly and indirectly respectively, to the TPT resonance.

## Results

### Experimental setup for the direct frequency comb optical frequency standard based on two-photon transitions of thermal atoms

**Figure 1:**
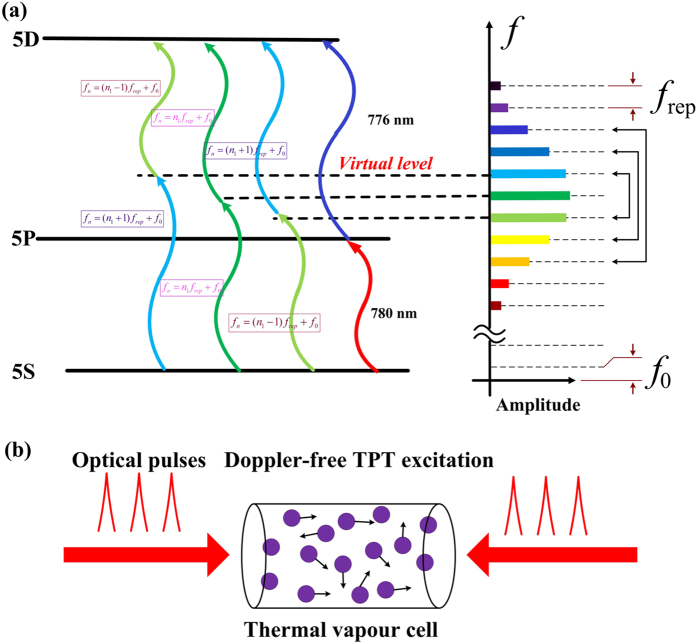
Three-level scheme used to describe the two-photon transitions excited by frequency comb. (**a**) Schematic of the joint participation of comb lines for TPT excitations. Once the comb is resonant with the TPT resonance, comb line pairs can jointly excite TPTs. The 5P state enables the occurrence of stepwise TPTs, involving the 5S → 5P (780 nm) and 5P → 5D (776 nm) transitions. (**b**) Doppler-free TPT excitations by two counter-propagating laser beams. For the elimination of Doppler-broadening TPTs background, various coherent quantum techniques are employed in this scheme (see methods).

**Figure 2:**
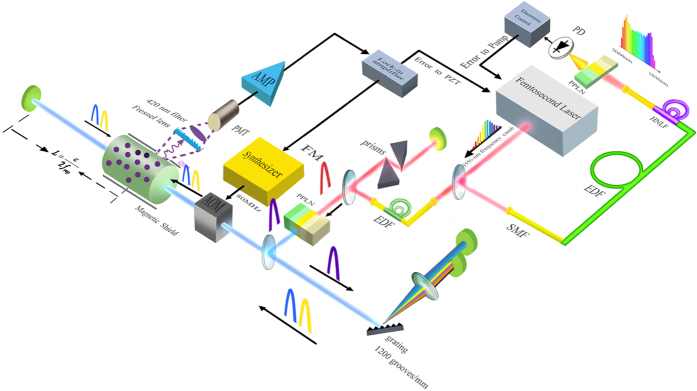
Schematic of the experimental set-up for the direct frequency comb optical frequency standard based on the TPTs of thermal atoms. The *f*-to-2*f* part is for detecting the carrier-envelop offset frequency *f*_0._ Other parts are used to generate the SH comb, excite the Doppler-free TPTs, and lock the comb to the TPTs. PZT, piezo-electric transducer; AMP, electronic amplifier; SMF, single-mode fibre; FM, frequency modulation; APD, avalanche photodiode; EDF, erbium-doped fibre; HNLF, highly nonlinear fibre.

The experimental setup for our direct frequency comb optical frequency standard based on the TPT of thermal Rb atoms is illustrated in Fig. 2. An Er: fibre femtosecond laser is used to generate the frequency comb centred at 1556 nm. One part of the comb is used for the stabilisation of *f*_0_. This part is firstly amplified to ~200 mW by an Er-doped fibre amplifier and then directed into an f-to-2f interferometer^32^, where *f*_0_ is generated by the self-referencing technique and precisely stabilised to the *f*_rep_ by tuning the pump power (see Methods). Another part is firstly amplified, and then dispersion compensated by a pair of silicon prisms to form the transform-limited pulses, so as to raise the second-harmonic-generation efficiency of the comb. The comb is then frequency doubled in a periodically poled lithium niobate (PPLN) crystal, resulting in 40 mW second-harmonic (SH) comb centred at 778 nm. TPTs excited by each unidirectional beam (Fig. 1b) give rise to a Doppler-broadening background, which blurs the Doppler-free signals^29^,^33^. Hence, we utilise the “split-pulse” technique^29^ (involving a grating, a lens and a pair of mirrors in Fig. 2) to eliminate the nonresonant type of TPT background. Besides, we greatly eliminate the stepwise (resonant) type of two-photon transition background by tuning the *f*_rep_ to destroy the resonant condition^34^. More details about the background elimination are described in Methods. After frequency shifted by an acousto-optic modulator (AOM, used for comb line locking), the SH comb is focused in the thermal ^87^Rb cell. The pulses are reflected back by a mirror placed at a distance of c/2*f*_rep_ (where c is the speed of light in the space) from the center of the cell, to ensure the two pulses collide temporally in the middle of the Rb cell. The Doppler-free TPTs take place in the centre of the cell when the returned pulses overlap with the counter-propagating pulses.

### Doppler-free two-photon transitions spectrum

**Figure 3:**
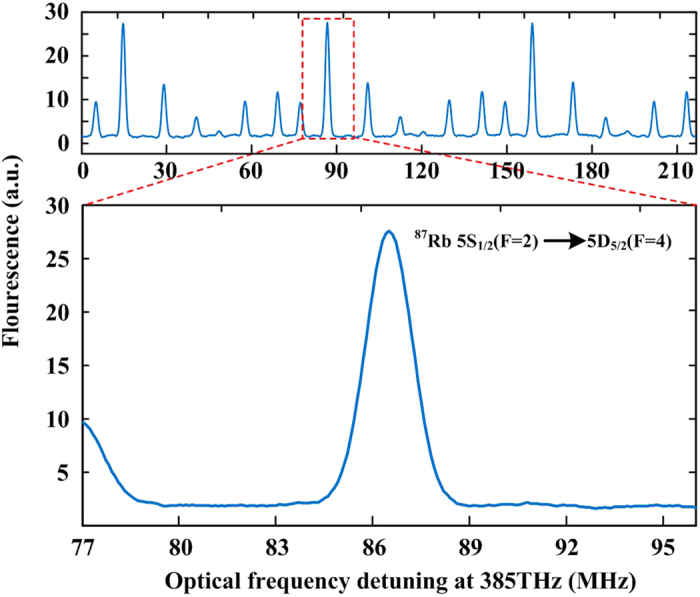
High quality TPTs spectrum of ^87^Rb with eliminated Doppler broadening background. (**a**) The observed spectrum of ^87^Rb Doppler-free TPTs directly excited by the frequency comb with the *f*_rep_ scanned around 144.198 MHz. (**b**) The 5S_1/2_ (F = 2) to 5D_5/2_(F = 4) TPT transition. This TPT has the highest SNR among all of the transitions, and is selected for the stabilization of the frequency comb.

The TPT signals are monitored by detecting the 420 nm fluorescence from cascade decay via the 6P state with a photomultiplier tube (PMT). By scanning the *f*_rep_, we can obtain the DFCS of the TPTs (Fig. 3a), which illustrates high-resolution 5S → 5D transition lines with a *f*_rep_ -scanning repetition period of 27 Hz and weak Doppler-broadening background below 5% intensity of the strongest transition line. Each of the TPT lines performs a linewidth of ~2 MHz in optical domain (Fig. 3b), which is mainly due to the 1.5-MHz residual Doppler broadening and 300-kHz transit time broadening with a laser beam waist diameter of ∼150 μm. The rest linewidth broadening is related to power broadening and beam misalignment. The Doppler effect can be suppressed to ~1 × 10^−3^ in thermal Rb atomic system.

The 5S_1/2_ (F = 2) → 5D_5/2_ (F = 4) transition is chosen as the optical frequency standard. Before the determination of this unperturbed two-photon transition frequency, the evaluation of systematic shifts needs to be investigated. These systematic shifts are presented in Table 1. The main systematic effect in the present work is light shift. This shift scales linearly with the average power of the laser, and can be corrected by performing measurements at different optical powers and extrapolating to zero. In our experimental conditions, this shift is about −47.3 kHz at 22-mW average power.

According to previous study^35^, a collisions shift about −181 ± 16 Hz is expected at the temperature of 60 ± 1 °C. This is difficult to quantify shifts from impurities in the vapour cell since the pressure of impurities is hardly changing with the temperature. We take a conservative upper limit for the pressure shift equal to 1 kHz. The smaller shifts including the relativistic shift, the black-body radiation shift and the collisions shift are also quantified. The Rb cell is placed in a specially designed μ-metal magnetic shield for reducing the influence of external fields, we estimate an upper limit of 703-Hz magnetic shift, providing 0.4 Gauss maximum residual magnetic field. In the locking process, the electronics induces approximately −1.6 kHz frequency shift.

Taking into account the evaluated systematic effects and 5-kHz statistical uncertainty related to the absolute measurement, the 5S_1/2_(F = 2) to 5D_5/2_(F = 4) two-photon transition is measured to be 385 284 566 373(10) kHz. This result is well consistent with previous measurements^27^,^35^. The accuracy of this frequency standard can reach 2.5 × 10^−11^.

### Frequency instability of the direct frequency comb optical frequency standard based on two-photon transitions of thermal ^87^Rb atoms

**Figure 4:**
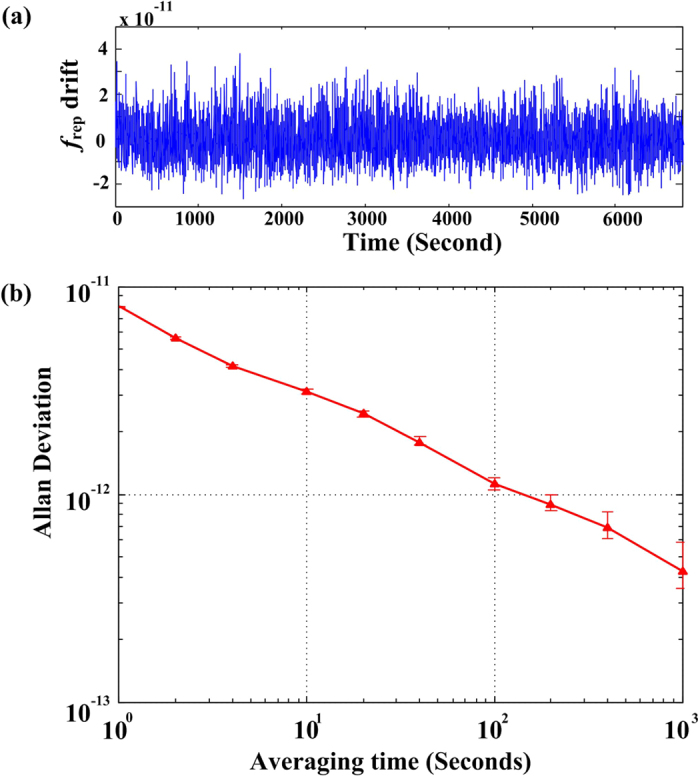
The stability of the TPT stabilised comb. (**a**) Time records of the fractional frequency drift of the stabilised repetition rate. (**b**) Fractional frequency instability of the stabilised repetition rate.

After getting the high-resolution TPT lines, the next step is to lock comb lines to the 5S_1/2_ (F = 2) → 5D_5/2_ (F = 4) transition, which has the maximum SNR among all the TPTs of ^87^Rb. For this purpose, an audio frequency signal is applied on the microwave driver (80 MHz) of the AOM. By detecting the phase signal from the 420 nm fluorescence, a digital lock-in-amplifier is employed to generate the error signal, and feeds it back into the actuator of the repetition rate (see Methods). With the *f*_0_ previously frequency locked to the *f*_rep_, the comb is fully stabilised to the TPT resonance. The curve in Fig. 4(a) shows the fractional frequency drifts of the fundamental repetition rate at 144 MHz, which is stabilised within 6 mHz. Figure 4(b) shows the measured fractional frequency instabilities in terms of overlapping Allan deviation. The instability reaches 8.0 × 10^−12^ and 4.3 × 10^−13^ for averaging times of 1 s and 1,000 s, respectively. The frequency instability of our system is mainly limited by the instability of the fibre laser, which induces frequency noise from the environment and the light shift caused by variations in the optical power.

## Discussion

The demonstrated optical frequency standard takes advantage of the narrow-linewidth optical comb to directly excite the narrow TPTs of thermal atoms, and then frequency locks the comb to these transitions. The optical comb enables the use of various coherent quantum techniques for significant Doppler-broadening elimination and SNR improvement^29^,^33^,^36^. Although the currently demonstrated system is still a tableful system, most parts are fibre-based components, and can be easily integrated into a small box. Moreover, the robustness of the whole system can be greatly improved by using an all polarization-maintaining, all fibre frequency comb^37^,^38^ as the probe laser. This is also our future work. The current stability demonstrated here is only a first step in this direction, it is a promising indicator that more advances can be made to achieve 10^−13^ instability with respect to the further reduction of Doppler-broadening and improvement of SNR.

This proof-of-concept system can also be expanded to other narrower TPTs, like the ultraviolet 1S-2S two-photon resonance with a natural linewidth of ~1 Hz in atomic hydrogen^16^, and can obtain a better stability and accuracy. When combining with the cold atomic system, it can be easy to realize 10^−15^ instability and uncertainty^39^, which is very close to the optical clock based on the ultrastable optical cavities, laser cooling and particle trapping techniques. Ultimately, the direct frequency comb optical frequency standards based on TPTs of thermal atoms are likely to benefit many practical applications, such as timing and synchronization systems, ultra-broadband and secure communications systems.

## Methods

### Elimination of the Doppler broadening background

We use a passive Er: fibre femtosecond pulse laser with a repetition frequency of ~144 MHz and an optical bandwidth of about 30 nm at a centre wavelength of 1,556 nm, as the probe laser and frequency standard output. In the observation of the Doppler-free Rb TPT transition directly excited by two counterpropagating laser beam, the undesired Doppler broadening transitions including direct TPTs (D-TPTs) and sequential TPTs (S-TPTs) are also happening at the same time and result in a Doppler-broadening background, and degrade the stability. Therefore, various coherent quantum techniques are utilized to estimate the Doppler-broadening background. As Fig. 2 shows, a 1200 grooves∕mm grating is used to disperse the beam. Two mirrors placed parallel to the Fourier plane are used to reflect >778 nm part and <778 nm part of the frequency comb spectrum. The <778-nm and >778-nm part are introduced by different time delays, therefore the two dispersed pulses cannot arrive at the vapour cell at the same time, so that they are inhibited to simultaneously excite the unidirectional D-TPTs.

For the S-TPTs Doppler-broadening background excited by one unidirectional beam, two of the comb lines are required to be resonant with the 5S → 5P (780 nm) and 5P → 5D (776 nm) transitions, respectively. When *f*_rep_ is a subharmonic of the frequency difference ∆*f* between the two-step 5S → 5P → 5D transitions, i.e., *f*_rep_ = ∆*f* /N (N is an integer), there are always some velocity groups of atoms to be excited by different mode pairs of the comb and contribute to the S-TPTs Doppler-broadening background. For the elimination of S-TPTs background, the *f*_rep_ is far detuned from the resonance values ∆*f* /N. More detailed information about these coherent quantum techniques can be found in ref. 34.

### Stabilisation of the carrier-envelop offset frequency

To detect the *f*_0_, we employ the conventional *f*-to-*2f* self-referencing technique, as shown in Fig. 2. The output of the femtosecond laser is amplified to 200 mW. The amplified pluses are injected into a highly nonlinear fibre (HNLF) to generate a supercontinuum spectrum. The spectrum around 2060 nm in the supercontinuum spectrum is doubled by second-harmonic generation in a PPLN. This generated 1030-nm light beats with the fundamental 1030-nm light in an avalanche photodiode (APD). The beating signal *f*_o_ at 40 MHz is then amplified and filtered, mixed with 1-GHz microwave, and divided by 32 in frequency. This signal is digitally phase-locked with a signal from direct digital synthesizer referenced to the *f*_rep_, by controlling the pump current of the fibre comb.

### Stabilisation of the repetition rate

Before the optical pulses enter the Rb cell, we firstly frequency shift them by 80 MHz in the AOM. For the phase locking, this 80-MHz signal is frequency modulated by an audio frequency (~780 Hz). After frequency-shifted, modulated, the counter-propagating pulses collide in the centre of the cell and excite the Doppler-free TPTs. We use a PMT to detect the 420-nm fluorescence from the cascaded 5D → 6P → 5S spontaneous radiation. The electronic signal from the PMT contains the phase information of the audio frequency, and is delivered into a digital lock-in-amplifier to generate the error signal. We feed it back into the intracavity piezoelectric transducer to stabilise the repetition rate.

## Additional Information

**How to cite this article**: Zhang, S. Y. *et al*. Direct frequency comb optical frequency standard based on two-photon transitions of thermal atoms. *Sci. Rep*. **5**, 15114; doi: 10.1038/srep15114 (2015).

## Figures and Tables

**Table 1:** Summary of the systematic frequency shifts.

Effects	Shifts at 778 nm
Light shift	−43.2 ± 3 kHz
Relativistic shift	−360 ± 1 Hz
Collisions shift	−181 ± 16 Hz
Collisions shift due to background gases	<1 kHz
Black-body radiation shift	−330 ± 4 Hz
Magnetic shift	<703 Hz
Electronic shifts	−1.6 ± 0.2 kHz
